# NRF2 activation induced by PML‐RARα promotes microRNA 125b‐1 expression and confers resistance to chemotherapy in acute promyelocytic leukemia

**DOI:** 10.1002/ctm2.418

**Published:** 2021-05-06

**Authors:** Xibao Yu, Ardalan Mansouri, Zhuandi Liu, Rili Gao, Kehan Li, Cunte Chen, Youxue Huang, Zheng Chen, Shaohua Chen, Yuhong Lu, Yangqiu Li, Chengwu Zeng, Yixin Zeng

**Affiliations:** ^1^ Department of Experimental Research Sun Yat‐Sen University Cancer Center, State Key Laboratory Oncology in South China Guangzhou China; ^2^ Key Laboratory for Regenerative Medicine of Ministry of Education Institute of Hematology, School of Medicine Jinan University Guangzhou China; ^3^ Department of Anatomy and Molecular Embryology Institute of Anatomy, Ruhr‐University Bochum Bochum Germany; ^4^ Department of Hematology First Affiliated Hospital, Jinan University Guangzhou China

Dear Editor,

Chromosomal translocation is a hallmark of acute myeloid leukemia (AML), often leading to gene rearrangements and expression of a fusion oncoprotein.^1^ In this work, we described the fusion oncoprotein promyelocytic leukemia‐retinoic acid receptor alpha (PML‐RARα) as a new mechanism for nuclear factor erythroid 2 (NF‐E2)‐related factor 2 (NRF2) activation during leukemogenesis and suggest that PML‐RARα‐induced NRF2/miR‐125b‐1 is uniformly important in reactive oxygen species (ROS) detoxification and the antileukemia response.

We have previously shown that PML‐RARα resulting from t(15;17) translocation, leads to aberrantly high expression of microRNA 125b‐1 (miR‐125b‐1) in acute promyelocytic leukemia (APL, M3 subtype of AML).^2^ In this study, we wanted to understand the mechanism by which PML‐RARα regulates miR‐125b‐1. Although the central role of fusion oncoprotein in the pathogenesis of AML has been recognized for a long time, and previous reports have suggested that activation of NRF2 mediates upregulation of miR‐125b‐1 in AML,^3^, ^4^ there is little known about the mechanism of NRF2 activation and how PML‐RARα regulates miR‐125b‐1. Similar to previous studies,^3^ NRF2 was expressed at high levels in primary APL samples compared to healthy individuals (HIs), as well as in non‐APL AML (Figures S1A‐S1C). Notably, we observed that NRF2 was predominantly located in the cytoplasm in HIs and those AML in CR (Complete Remission); however, it was mainly in the nucleus in primary APL and non‐APL AML (Figures 1A and S1D). Importantly, PML‐RARα could significantly enhance the protein level of NRF2 (Figure 1B) without effect on its mRNA (Figure S1E). Analysis of NRF2 in the cytosolic and nuclear fractions indicated that PML‐RARα induced constitutive nuclear levels of NRF2 (Figures 1C, 1D, and S1F). Moreover, PML‐RARα could inhibit the degradation of NRF2 (Figure 1E). These results are in line with previously published data implying that PML‐RARα is correlated with NRF2 degradation.^5^ These results indicate that NRF2 activation may be associated with the development of APL, and PML‐RARα may increase miR‐125b‐1 expression by inducing constitutive nuclear levels of NRF2.

**FIGURE 1:**
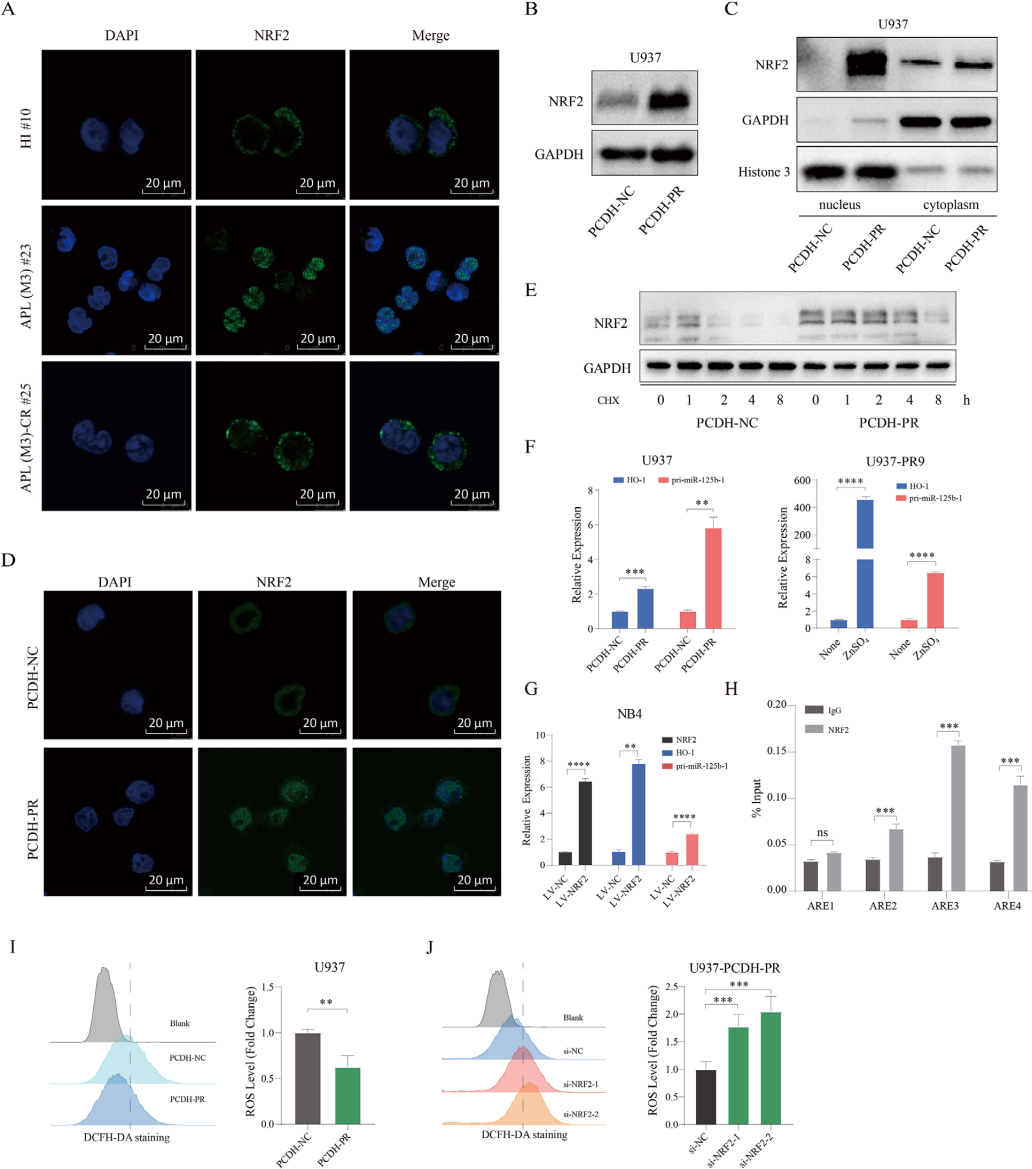
PML‐RARα activates the NRF2/miR‐125b‐1 antioxidant program. (A) Immunofluorescence microscopy after staining with NRF2 (green) and DAPI (blue) demonstrated NRF2 expression in HI, APL (M3) and APL (M3)‐CR cells. Scale bars, 20 μm. (B and C) U937‐PCDH‐NC and U937‐PCDH‐PR cells were analyzed for NRF2 in whole cell lysates or cytosolic/nuclear fractions by Western blot. Blots are representative of at least three independent experiments. (D) Immunofluorescence microscopy of NRF2 in stable constitutive express *PML‐RARA* (U937‐PCDH‐PR) or negative control (U937‐PCDH‐NC) cells. A representative image of three independent experiments is shown. Scale bars, 20 μm. (E) Western blot analysis of NRF2 in U937‐PCDH‐NC and U937‐PCDH‐PR cells treated with 20 μM cycloheximide at different time points. (F) qRT‐PCR analysis of HO‐1 and pri‐miR‐125b‐1 in U937‐PCDH‐PR or U937‐PR9 (a zinc‐inducible *PML‐RARA* cell line derived from U937) cells. *p* values were obtained by unpaired Student's *t* test. (G) qRT‐PCR analysis of NRF2, HO‐1 and pri‐miR‐125b‐1 in NRF2‐overexpressing NB4 cells. (H) ChIP‐qPCR analysis of NB4 cells demonstrated that immunoprecipitation (IP) with an anti‐NRF2 antibody resulted in enrichment of the three putative binding sites compared with IP with a control immunoglobulin. (I) The ROS levels in U937‐PCDH‐NC and U937‐PCDH‐PR cells were measured by a DCFH‐DA probe. (J) ROS levels in U937‐PCDH‐PR cells were analyzed after knocking down the *NRF2* gene. Data are presented as the means ± SD from at least three independent experiments. Detailed methods are available in Methods in the Supporting Information

Notably, we observed that PML‐RARα could promote expression of the primary transcript of *miR‐125b‐1* (pri‐miR‐125b‐1) and the NRF2 target gene *HO‐1*, *MT1X*, and *MT2* (Figures 1F, S2A, and S2B). A recent study reported PML‐RARα decreased NRF2 activity upon zinc treatment;^6^ however, PML‐RARα increased HO‐1 levels in our model even in the presence of zinc (Figure S2C). Knocking down *NRF2* in PML‐RARα‐expressing cells could markedly inhibit pri‐miR‐125b‐1 expression (Figures S2D and S2E). In contrast, overexpression of NRF2 or downexpression of KEAP1 (a negative regulator of NRF2) could increase the abundance of pri‐miR‐125b‐1 (Figures 1G and S2F). The above results indicated that PML‐RARα promotes miR‐125b‐1 expression in an NRF2‐dependent manner. Next, we sought to test whether NRF2 could directly bind to the *miR‐125b‐1* promoter by chromatin immunoprecipitation (ChIP). As shown in Figure 1H, NRF2 was found to directly bind the antioxidant response elements (AREs) within the promoter region of *miR‐125b‐1*, demonstrating that *miR‐125b‐1* is a direct target of NRF2 in APL. Because NRF2 signaling is a critical survival pathway that regulates cellular oxidative stress responses,^7^ we next determined whether ROS levels could be suppressed by NRF2. There were lower ROS levels in PML‐RARα expressing cells (Figures 1I and S3A), and downregulation of NRF2 expression could increase ROS levels (Figures 1J, S3B, and S3C). These observations demonstrated that NRF2 is responsible for the elevated miR‐125b‐1 expression and the activated antioxidant program in APL cells.

Arsenic trioxide (ATO) has been used as leukemia therapies to target PML‐RARα, and recent finding also suggests the potential benefit of ATO in treating p53‐mutated tumor.^8^ Here, we found that ATO treatment further induced NRF2 and miR‐125b‐1 expression (Figures 2A‐2C). Loss of NRF2 significantly inhibited pri‐miR‐125b‐1 expression in ATO‐treated NB4 cells (Figure 2D). Accordingly, ChIP assays showed that NRF2 could bind to all four AREs in the *miR‐125b‐1* promoter in these cells (Figure 2E). Because many chemotherapeutic drugs can induce NRF2 nuclear accumulation via oxidative stress,^9^ we sought to determine whether elevated ROS levels could be responsible for NRF2 activation. Indeed, pretreatment with the antioxidant scavenger N‐acetyl‐cysteine (NAC) blocked ATO‐induced ROS production, expression of HO‐1, and pri‐miR‐125b‐1 (Figures 2F and 2G). Besides, other chemotherapeutic agents (AraC and MG132) could also induce the generation of ROS and pri‐miR‐125b‐1 expression (Figures S4A and S4B). These findings indicated that miR‐125b‐1 is a downstream NRF2 target gene whose transcription is activated in response to chemotherapy in a ROS‐dependent mechanism.

**FIGURE 2:**
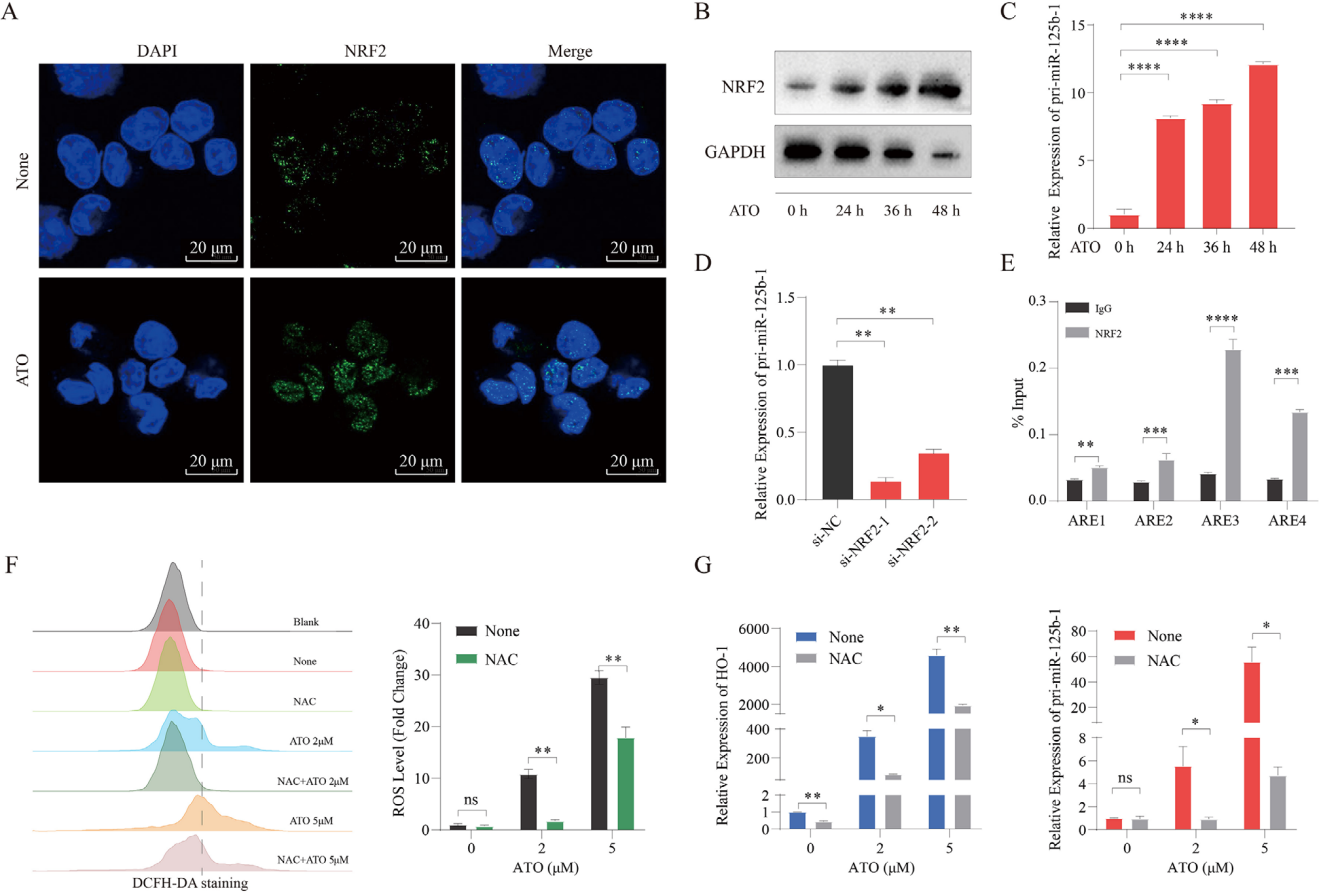
Chemotherapeutic drug treatment‐induced activation of NRF2 leads to upregulation of miR‐125b‐1. (A) Immunofluorescence microscopy after staining with NRF2 (green) and DAPI (blue) demonstrated NRF2 expression in NB4 cells treated with 2 μM ATO for 48 h. None, untreated; Scale bars, 20 μm. (B) Western blot analysis of NRF2 in NB4 cells treated with 2 μM ATO for the indicated times. GAPDH served as loading control. (C) qRT‐PCR analysis of pri‐miR‐125b‐1 in NB4 cells treated with 2 μM ATO for the indicated times. (D) qRT‐PCR analysis of pri‐miR‐125b‐1 in NB4 cells transduced with the indicated siRNA and treated for 48 h with 2 μM ATO. (E) ChIP‐qPCR analysis of NB4 cells treated with 2 μM ATO for 48 h demonstrated that immunoprecipitation (IP) with an anti‐NRF2 antibody resulted in enrichment of the four putative binding sites compared with IP with control immunoglobulin. (F) ROS levels were assessed by flow cytometric analysis. The NB4 cells were pretreated with 5 mM NAC for 4 h and then treated with 2 μM or 5 μM ATO for 24 h. (G) qRT‐PCR analysis of HO‐1 and pri‐miR‐125b‐1 in NB4 cells. The NB4 cells were pretreated with 5 mM NAC for 4 h and then treated with 2 μM or 5 μM ATO for 24 h. The graph represents the mean and SD of three independent experiments. Results were normalized by the respective controls

Chemotherapy‐mediated cytotoxicity has been associated with ROS generation and the induction of apoptosis. The fact that chemotherapy sustainably increases NRF2/miR‐125b‐1 levels prompted us to test the impact of NRF2/miR‐125b‐1 on response to chemotherapy. We found that ROS suppression by NAC hindered chemotherapeutic drug‐induced apoptosis (Figure S4C), thus demonstrating that elevated ROS is responsible for these cytotoxic responses. We observed that NRF2 attenuated ATO‐induced apoptosis in APL cells (Figures 3A and 3B). Importantly, overexpression of miR‐125b strongly reduced responsiveness to ATO treatment (Figures 3C‐3E), as well as AraC and MG132 (Figures S5A‐S5D). Besides, miR‐125b inhibited the suppression of colony formation by chemotherapeutic drugs (Figure 3F). Consistently, miR‐125b impaired ROS production induced by chemotherapeutic drugs (Figure 3G). Also, ROS‐generating NADPH oxidase (NOX2 complex) gene expression was decreased in miR‐125b‐overexpressing NB4 cells and APL patients, and overexpression of miR‐125b could increase the level of NRF2 in APL cells (Figures 4A and S6A‐S6C). Furthermore, we found that several pro‐apoptotic known targets (BAK1, BBC3, and BMF) of miR‐125b were downregulated by miR‐125b (Figure 4B).^2^, ^10^ In particular, miR‐125b repressed BAK1 expression in cells with or without ATO treatment (Figure 4C). BAK1 depletion mimicked the effects of miR‐125b (Figures 4D‐4F), and overexpression of BAK1 increased the level of ROS (Figure S6D), showing that BAK1 is a functional target gene of miR‐125b. Overall, these findings demonstrated that miR‐125b possesses a critical antioxidant effect and therefore protects APL cells from chemotherapy‐induced cytotoxicity.

**FIGURE 3:**
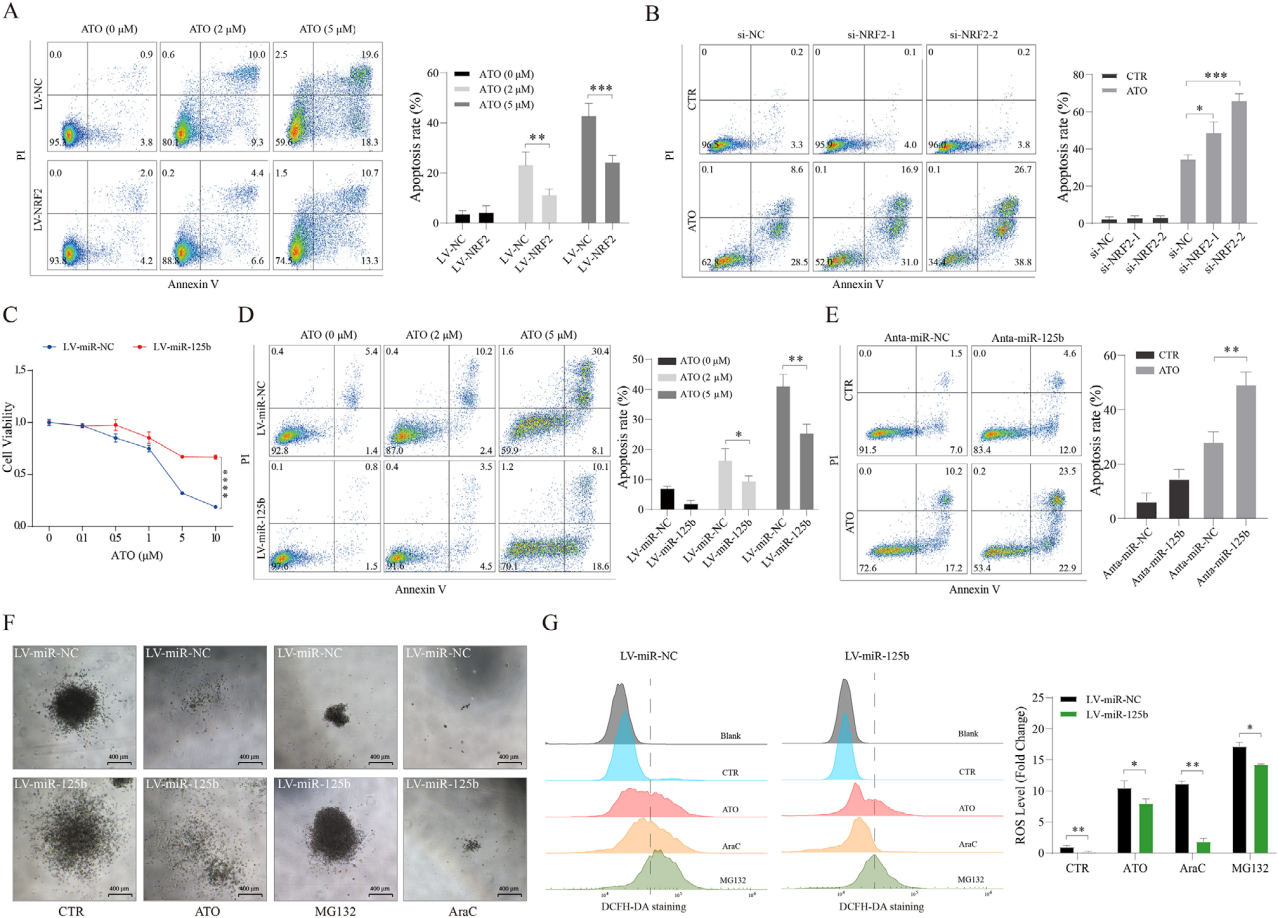
NRF2/miR‐125b‐1 protects against chemotherapy‐induced apoptosis in APL. (A) Flow cytometric analysis of the apoptosis of NB4‐LV‐NC and NB4‐LV‐NRF2 cells treated with 2 μM or 5 μM ATO for 24 h. (B) NB4 cells were transfected with control siRNA or NRF2 siRNA followed by a 48 h ATO (2 μM) treatment. (C) Dose‐response curves from cell viability assays of the NB4‐LV‐miR‐NC and NB4‐LV‐miR‐125b cells treated with the indicated concentration of ATO for 48 h. (D) NB4‐LV‐ NC and NB4‐LV‐miR‐125b cells were treated with 2 μM or 5 μM ATO for 24 h. (E) Treatment of NB4 cells transduced with either miR‐125b antagomir and miRNA antagomir control and treated with 2 μM ATO (48 h). (F) Morphology of colonies formed by NB4‐LV‐miR‐NC and NB4‐LV‐miR‐125b cells treated with 2 μM ATO, 0.1 μM MG132, and 0.1 μM AraC. Scale bars, 400 μm. (G) ROS levels in NB4‐LV‐miR‐NC and NB4‐LV‐miR‐125b cells treated with ATO (5 μM), MG132 (1 μM), or AraC (1 μM) for 24 h. A representative image of three independent experiments is shown. Values are derived from three independent experiments data are reported as mean ± SD

**FIGURE 4:**
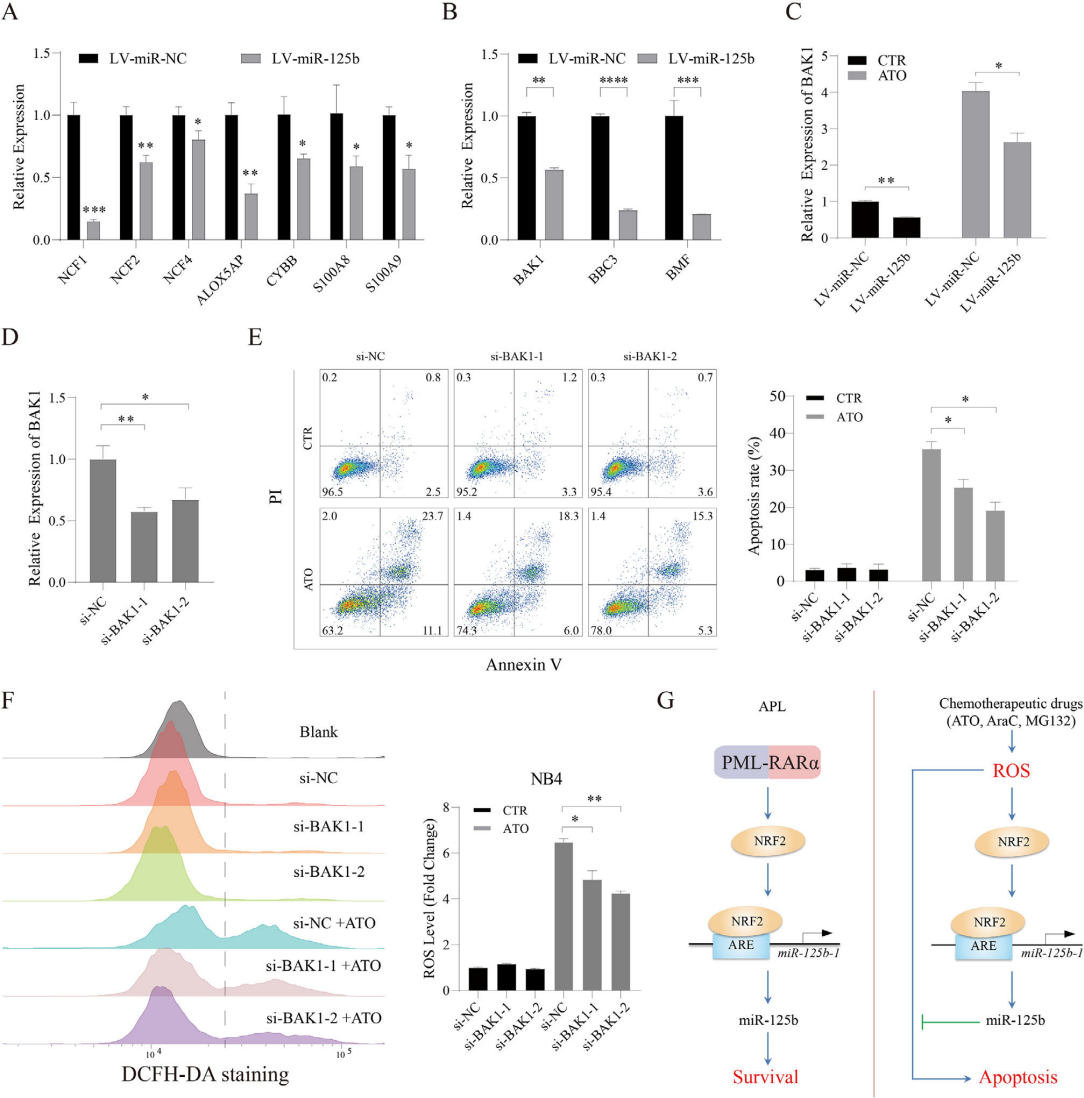
BAK1 is a functional target of miR‐125b in the response of APL cells to chemotherapy. (A) qRT‐PCR analysis of ROS production‐related enzyme genes in NB4‐LV‐miR‐NC and NB4‐LV‐miR‐125b cells. (B) Changes of BMF, BAK1, and BBC3 expression in NB4 cells upon miR‐125b overexpression by qRT‐PCR. (C) qRT‐PCR analysis of BAK1 in NB4‐LV‐miR‐NC and NB4‐LV‐miR‐125b cells treated with 2 μM ATO for 48 h. Knockdown efficiency (D), apoptosis rate (E), and ROS levels (F) in NB4 cells were analyzed after knocking down the BAK1 gene followed by ATO (2 μM) treatment. (G) Proposed model depicting regulation and role of NRF2/miR‐125b‐1 in APL

Together, our data demonstrate that miR‐125b‐1 expression is likely due to PML‐RARα‐mediated NRF2 activity, thus explaining the elevated miR‐125b‐1 in primary APL samples. Moreover, we showed that NRF2/miR‐125b‐1 plays an important role in ROS detoxification and the resistance to chemotherapy (Figure 4G). Our study provides evidence and a rationale for targeting NRF2/miR‐125b‐1 in the development of therapeutics for improving chemotherapy.

## CONFLICT OF INTEREST

The authors declare that there is no conflict of interest.

## AVAILABILITY OF DATA AND MATERIALS

The authenticity of this article has been validated by uploading the key raw data onto the Research Data Deposit public platform (www.researchdata.org.cn), with the approval RDD number as RDDB2021001607.

## AUTHOR CONTRIBUTIONS

Xibao Yu, Ardalan Mansouri, Zhuandi Liu, and Rili Gao performed the experiments, wrote the paper, and analyzed the data. Kehan Li, Cunte Chen, Youxue Huang, Zheng Chen, and Shaohua Chen helped analyze the data. Yuhong Lu provided primary cells and patient information. Chengwu Zeng, Yangqiu Li, and Yixin Zeng designed the study and wrote the manuscript. All authors read and approved the final manuscript.

## ETHICS APPROVAL AND CONSENT TO PARTICIPATE

This study was approved by the ethics committee of the affiliated hospitals of Jinan University. Written informed consent was obtained from all patients.

## Supporting information



Supporting Information
