# Supplementary material for: NRF2 activation induced by PML‐RARα promotes microRNA 125b‐1 expression and confers resistance to chemotherapy in acute promyelocytic leukemia
Source: Clin Transl Med. 2021 May 6;11(5):e418. doi: 10.1002/ctm2.418 (PMC8101532; doi:10.1002/ctm2.418)
Supplement: Supplementary file 1 — Supporting Information [file CTM2-11-e418-s001.doc]

**Supporting Information**

**NRF2 activation induced by PML-RARα promotes microRNA 125b-1 expression and confers resistance to chemotherapy in acute promyelocytic leukemia**

Xibao Yu ^1, 2, #^, Ardalan Mansouri ^2, 3, #^, Zhuandi Liu ^2, #^, Rili Gao ^2^, Kehan Li ^2^, Cunte Chen ^2^, Youxue Huang ^2^, Zheng Chen ^2^, Shaohua Chen ^2^, Yuhong Lu ^4^, Yangqiu Li ^2, †^, Chengwu Zeng ^2, †^, Yixin Zeng ^1, †^

^1^ Department of Experimental Research, Sun Yat-sen University Cancer Center, State Key Laboratory Oncology in South China, Guangzhou 510060, China

^2^ Key Laboratory for Regenerative Medicine of Ministry of Education, Institute of Hematology, School of Medicine, Jinan University, Guangzhou 510632, China.

^3^ Department of Anatomy and Molecular Embryology, Institute of Anatomy, Ruhr-University Bochum, 44801 Bochum, Germany

^4^ Department of Hematology, First Affiliated Hospital, Jinan University, Guangzhou, China.

^#^These authors contributed equally to this work.

**^†^Correspondence**

Yangqiu Li and Chengwu Zeng, Jinan University, No.601, West Huangpu Avenue, Guangzhou 510632, Guangdong, China.

Yixin Zeng, Department of Experimental Research, SunYat-sen University Cancer Center, State Key Laboratory Oncology in South China, Guangzhou 510060, China.

E-mail:

CWZ: [bio-zcw@163.com](mailto:bio-zcw@163.com), [zengcw@jnu.edu.cn](mailto:zengcw@jnu.edu.cn); YQL: [yangqiuli@hotmail.com](mailto:yangqiuli@hotmail.com); YXZ: zengyx@sysucc.org.cn;

**METHODS**

**Patients and samples**

32 peripheral blood mononuclear cell (PBMC) samples and 8 CD34^+^ cells (sorted from cord blood samples by CD34 microBeads) were obtained from the First Affiliated Hospital of Jinan University and enrolled in this study (Table 1). All samples were obtained with consent, and this study was approved by the Ethics Committee of the Affiliated Hospitals of Jinan University.

**Cell culture and reagents**

NB4, U937, and U937-PR9 (a zinc-inducible *PML-RARA* cell line derived from U937) cells were cultured in RPMI 1640 containing 10% fetal bovine serum. The cells were cultured in a humidified atmosphere containing 5% CO_2_ at 37°C. ATO was dissolved in 1 N NaOH and further diluted to 10 mM in PBS . N-acetyl-L-cysteine (NAC) and cycloheximide (CHX) was purchased from Selleck Chemicals.

**Plasmid construction and virus infection**

The long isoform of the *PML-RARA* fusion gene was PCR-amplified from the pSG5-*PML-RARA* plasmid and BAK1 gene was amplified from cDNA of HI. Then, the PCR product was purified and cloned into the pCDH-CMV-MCS-EF1-Puro eukaryotic expression vector (pCDH-*PML-RARΑ*-Puro, PCDH-PR; pCDH-BAK1-Puro, PCDH-BAK1).

Lentiviral vectors expressing RNAi specific for the KEAP1 gene and a scrambled sequence were designed and constructed. The following sequences were used: 5’-GTGGCGAATGATCACAGCAAT-3’ (shKEAP1-1), 5’-GCAAGGACTACCTGGTCAAGA-3’ (shKEAP1-2), 5’-GCTCCTACTGTGATGTGAAAT-3’ (shNRF2) and 5’-GCGATGTGGCGAACTGACA-3’ (shNC). Pairs of complementary oligonucleotides with these sequences were synthesized, annealed and cloned into the lentiviral plasmid vector (plko.1-Puro).

A lentivirus carrying pCDH-Puro or plko.1-Puro was produced by transfecting HEK293T with standard packaging vectors using lipofectamine 3000 (Life Technologies). Viral supernatants were harvested 48 h and 72 h after transfection. The Lentivirus Concentration Kit (iGene Biotechnology, China) was used to precipitate the virus. A total of 1x10^4^ NB4 or U937 cells were incubated with concentrated virus supplemented with 8 mg/mL polybrene (Sigma-Aldrich, USA). Positively infected cells were selected by 5 μg/mL puromycin for 5 days.

The LV-NRF2-EGFP and LV-miR-125b-puro lentiviruses were purchased from Genechem. The lentivirus was added according to the multiplicity of infection (MOI) for NB4 cells (MOI = 30). For NB4-LV-NRF2-EGFP cells, GFP-positive cells were sorted by fluorescence activated cell sorting (FACS) 48 h after infection and plated in 48-well plates. For NB4-miR-125b-puro cells, positively infected cells were selected with 5 μg/mL puromycin for 5 days.

**RNA interference**

NB4 and U937-PCDH-PR (a U937 subclone stably transfected with *PML-RARA*), cells were transfected using the Neon® Transfection System (Invitrogen, USA) with 100 pmol of oligonucleotides in 10 μl reactions. Transfection was performed as described previously . The siRNA sequences targeting NRF2 were as follows:

si-NRF2-1, 5’- AAGAGUAUGAGCUGGAAAAAC-3’ and si-NRF2-2, 5’- GCUUUUGGCGCAGACAUUC-3’. miR-125b-5p antagomir (Anta-miR-125b) and miRNA antagomir control (Anta -miR-NC) were purchased from RiboBio (Guangzhou, China).

**Quantitative real-time PCR analysis**

RNA was extracted with TRIzol reagent (Invitrogen, USA), and first-strand cDNA was then generated using High-Capacity cDNA Reverse Transcription Kits (Applied Biosystems, USA) following the manufacturer’s instructions. qRT-PCR was performed with SYBR Green (TIANGEN, China) according to the manufacturer’s instructions. PCR was performed as described previously . Gene expression levels were normalized to an *ACTB* internal control. Primers are listed in Table 2. The qRT-PCR program was as follows: 95°C for 15 min followed by 40 cycles at 95 °C for 10 s and 60 °C for 30 s.

**Western blotting**

For preparation of whole cell extracts, treated cells were washed twice in PBS and lysed on ice for 20 min in RIPA buffer with protease inhibitors. Nuclear and cytosolic extracts were prepared using the Cytoplasmic and Nuclear Fractionation kit (SC-003, Invent) according to the manufacturer’s protocol. Protein extracts were separated by SDS-PAGE and then transferred to a polyvinylidene fluoride membrane. The blots were incubated with anti-NRF2 (SAB2701989, Sigma-Aldrich), anti-Histone H3 (4499S, CST), and anti-GAPDH (5174S, CST) antibodies. Chemiluminescent reactions were performed using the Immobilon™ Western Chemiluminescent HRP Substrate (Millipore, USA).

**Immunofluorescence**

Cells were collected onto slides and fixed in 4% formaldehyde for 10 min at room temperature followed by permeabilization with 0.1% Triton X-100 for 10 min. Fixed cells were blocked for 1 h with Blocking Buffer (Beyotime, China) and then incubated overnight with the anti-NRF2 antibody. The cells were washed 3 times for 5 min with PBS and then incubated for 1 h with Alexa fluor 488-labeled goat anti-rabbit IgG (Beyotime, China). Subsequently, the cell nuclei were stained with DAPI. The stained cells were observed by fluorescence microscopy (Leica DM6000B; Leica, Germany).

**Chromatin immunoprecipitation assay**

Chromatin immunoprecipitation (ChIP) assays were performed by following the EZ-ChIP™# 17-371 (Millipore, USA) instruction manual. Anti-NRF2 antibody or nonimmune rabbit IgG control (sc-2027, Santa Cruz) was used in the ChIP assays, and ChIP-qPCR primers (Table 2) were designed as previously described .

**ROS assay and apoptosis analysis**

ROS levels were measured using the oxidation-sensitive fluorescent probe 2, 7-dichlorofluorescein diacetate (DCFH-DA) (Beyotime, China) as a substrate. Briefly, after treatment, cells were stained with 5 μM DCFH-DA in PBS for 20 min in the dark, and the cells were washed three times with PBS. Flow cytometry was then performed at an excitation wavelength of 488 nm and an of emission wavelength 525 nm. Apoptosis was detected by staining with the Annexin-V-APC/PI Apoptosis Detection Kit (MultiSciences, China). Analysis was performed by flow cytometry using the manufacturer's protocol.

**Colony forming cell assay**

Colony forming cell assays were performed by plating NB4-LV-miR-NC or NB4-LV-miR-125b cells in methylcellulose media (R&D, USA) containing 1% methylcellulose, 10% FBS, and chemotherapeutic drugs according to the user manual. Cells were incubated at 37°C in 5% CO_2_, and microphotographs were taken eight days’ post plating with microscopy (Leica DM6000B; Leica, Germany).

**Cell viability analysis**

To assess cell viability, cell counting kit-8 (CCK-8) (Dojindo, Japan) was used according to the manufacturer's protocol. Briefly, NB4-LV-miR-NC and NB4-LV-miR-125b cells were plated at a density of 3,000 cells/well in 96-well plates and cultured in RPMI 1640 medium containing 10% FBS and respective chemotherapeutic drugs. CCK-8 reagent was added to the wells at the end of the experiment. After incubation at 37°C for 4 h, the absorbance of each well was determined using a microplate reader at 450 nm. Medium without cells was used as blank. After calculation, cell viability and was normalized to corresponding untreated controls.

**Statistical analysis**

Data are expressed as the mean ± SD of three independent experiments. Statistical analysis was executed using GraphPad Prism 8 software. The significance of differences between groups was determined by the Student’s *t* test (unpaired and two-tailed). A *P* value <0.05 was considered significant. *, *P* < 0.05, **, *P* < 0.01, ***, *P* < 0.001, and ****, *P* < 0.0001.

**References**

[1] L. Berthoux, G.J. Towers, C. Gurer, P. Salomoni, P.P. Pandolfi, J. Luban, As(2)O(3) enhances retroviral reverse transcription and counteracts Ref1 antiviral activity, J Virol 77 (2003) 3167-3180.

[2] Y. Wu, Y. Hu, X. Yu, Y. Zhang, X. Huang, S. Chen, Y. Li, C. Zeng, TAL1 mediates imatinib-induced CML cell apoptosis via the PTEN/PI3K/AKT pathway, Biochem Biophys Res Commun 519 (2019) 234-239.

[3] X. Yu, Y. Hu, Y. Wu, C. Fang, J. Lai, S. Chen, Y. Li, C. Zeng, Y. Zeng, The c-Myc-regulated miR-17-92 cluster mediates ATRA-induced APL cell differentiation, Asia Pac J Clin Oncol 15 (2019) 364-370.

[4] C. Zeng, S. Liu, S. Lu, X. Yu, J. Lai, Y. Wu, S. Chen, L. Wang, Z. Yu, G. Luo, Y. Li, The c-Myc-regulated lncRNA NEAT1 and paraspeckles modulate imatinib-induced apoptosis in CML cells, Mol Cancer 17 (2018) 130.

[5] X. Wang, S. Lu, Y. Xiao, L. Xu, L. Zhou, J. Hu, B. Li, C. Zeng, Y. Li, Alteration of gene expression profile in CD3(+) T-cells after downregulating MALT1, Immunotargets Ther 7 (2018) 77-81.

[6] C. Zeng, Y. Xu, L. Xu, X. Yu, J. Cheng, L. Yang, S. Chen, Y. Li, Inhibition of long non-coding RNA NEAT1 impairs myeloid differentiation in acute promyelocytic leukemia cells, BMC Cancer 14 (2014) 693.

[7] N.M. Shah, L. Zaitseva, K.M. Bowles, D.J. MacEwan, S.A. Rushworth, NRF2-driven miR-125B1 and miR-29B1 transcriptional regulation controls a novel anti-apoptotic miRNA regulatory network for AML survival, Cell Death Differ 22 (2015) 654-664.

**Table 1：**Clinical information relevant to samples

| **ID** | **group** | **ages** | **gender** | **Used in Figure** |
| --- | --- | --- | --- | --- |
| 1 | HI | 43 | F | S1A |
| 2 | HI | 37 | M | S1A |
| 3 | HI | 49 | F | S1A |
| 4 | HI | 39 | F | S1A |
| 5 | HI | 27 | M | S1A |
| 6 | HI | 31 | M | S1A |
| 7 | HI | 36 | M | S1A |
| 8 | HI | 50 | M | S1A |
| 9 | HI | 71 | F | S1A |
| 10 | HI | 71 | M | 1A |
| 11 | HI | 36 | M | 1A, NOT SHOWN |
| 12 | HI | 56 | M | 1A, NOT SHOWN |
| 13 | HI | 37 | F | 1A, NOT SHOWN |
| 14 | HI | 37 | F | S1D |
| 15 | APL | 48 | M | S1A |
| 16 | APL | 69 | F | S1A |
| 17 | APL | 64 | M | S1A |
| 18 | APL | 53 | F | S1A |
| 19 | APL | 30 | M | S1A |
| 20 | APL | 30 | M | S1A |
| 21 | APL | 27 | F | S1A |
| 22 | APL | 48 | M | S1A |
| 23 | APL | 48 | F | 1A |
| 24 | APL | 42 | M | S1D |
| 25 | APL-CR | 25 | M | 1A |
| 26 | APL-CR | 15 | F | 1A, NOT SHOWN |
| 27 | non-M3 AML | 12 | M | S1D |
| 28 | non-M3 AML | 23 | M | S1D, NOT SHOWN |
| 29 | non-M3 AML | 82 | M | S1D, NOT SHOWN |
| 30 | non-M3 AML | 60 | M | S1D, NOT SHOWN |
| 31 | non-M3 AML-CR | 47 | F | S1D |
| 32 | non-M3 AML-CR | 27 | F | S1D, NOT SHOWN |
| 33 | CD34^+^ | 0 | M | S1A |
| 34 | CD34^+^ | 0 | M | S1A |
| 35 | CD34^+^ | 0 | M | S1A |
| 36 | CD34^+^ | 0 | M | S1A |
| 37 | CD34^+^ | 0 | F | S1A |
| 38 | CD34^+^ | 0 | F | S1A |
| 39 | CD34^+^ | 0 | F | S1A |
| 40 | CD34^+^ | 0 | F | S1A |

**Table 2：The sequences of primers**

| Gene target | Sense (5' -> 3') | Antisense (5' -> 3') | |
| --- | --- | --- | --- |
| *ACTB* | TTGTTACAGGAAGTCCCTTGCC | | ATGCTATCACCTCCCCTGTGTG |
| *NRF2* | GAGAGCCCAGTCTTCATTGC | | TTGGCTTCTGGACTTGGAAC |
| *HO-1* | CCAGGCAGAGAATGCTGAGTTC | | AAGACTGGGCTCTCCTTGTTGC |
| pri-*miR-125b-1* | CCATACCACCTGTTTGTTGCATCT | | CTGAGAGGAGCGCAACAATGT |
| *KEAP1* | CAACTTCGCTGAGCAGATTGGC | | TGATGAGGGTCACCAGTTGGCA |
| *MT1X* | CTGCTTCTCCTTGCCTCGAA | | TGTCTGACGTCCCTTTGCAG |
| *MT2* | ATCCCAACTGCTCCTGCGCCG | | CAGCAGCTGCACTTGTCCGACG |
| *BAK1* | GCTCCCAACCCATTCACTAC | | TCCCTACTCCTTTTCCCTGA |
| *BMF* | CAGTGGCAACATCAAGCAGAGG | | GCAAGGTTGTGCAGGAAGAGGA |
| *BBC3* | ACGACCTCAACGCACAGTACGA | | CCTAATTGGGCTCCATCTCGGG |
| *miR-125b-1-ARE1* | ATGTTTCCAAACCAGGCTGA | | CTAACACTGCAGGCTCACCA |
| *miR-125b-1-ARE2* | CAGAGCCAGCTGTCAATGAA | | CCAGAATGGGAGAAATGGAG |
| *miR-125b-1-ARE3* | GTTGAGGCCTCTCCAGTGTC | | GCCACCAAAAATGAAAGGAA |
| *miR-125b-1-ARE4* | TGAGCAAGGTAGATGGCTGG | | AGTTGTCTTGAAGGTGGGGG |
| *PML-RARA*-CDS | GCTCTAGAATGGAGCCTGCACCCGCCCGATC | | CGGAATTCTCACGGGGAGTGGGTGGCCGGGC |
| *BAK1*-CDS | GCTCTAGAATGGCTTCGGGGCAAGGCCC | | CGGAATTCTCATGATTTGAAGAATCTTC |

**Supplementary Figures**

Figure S1

**
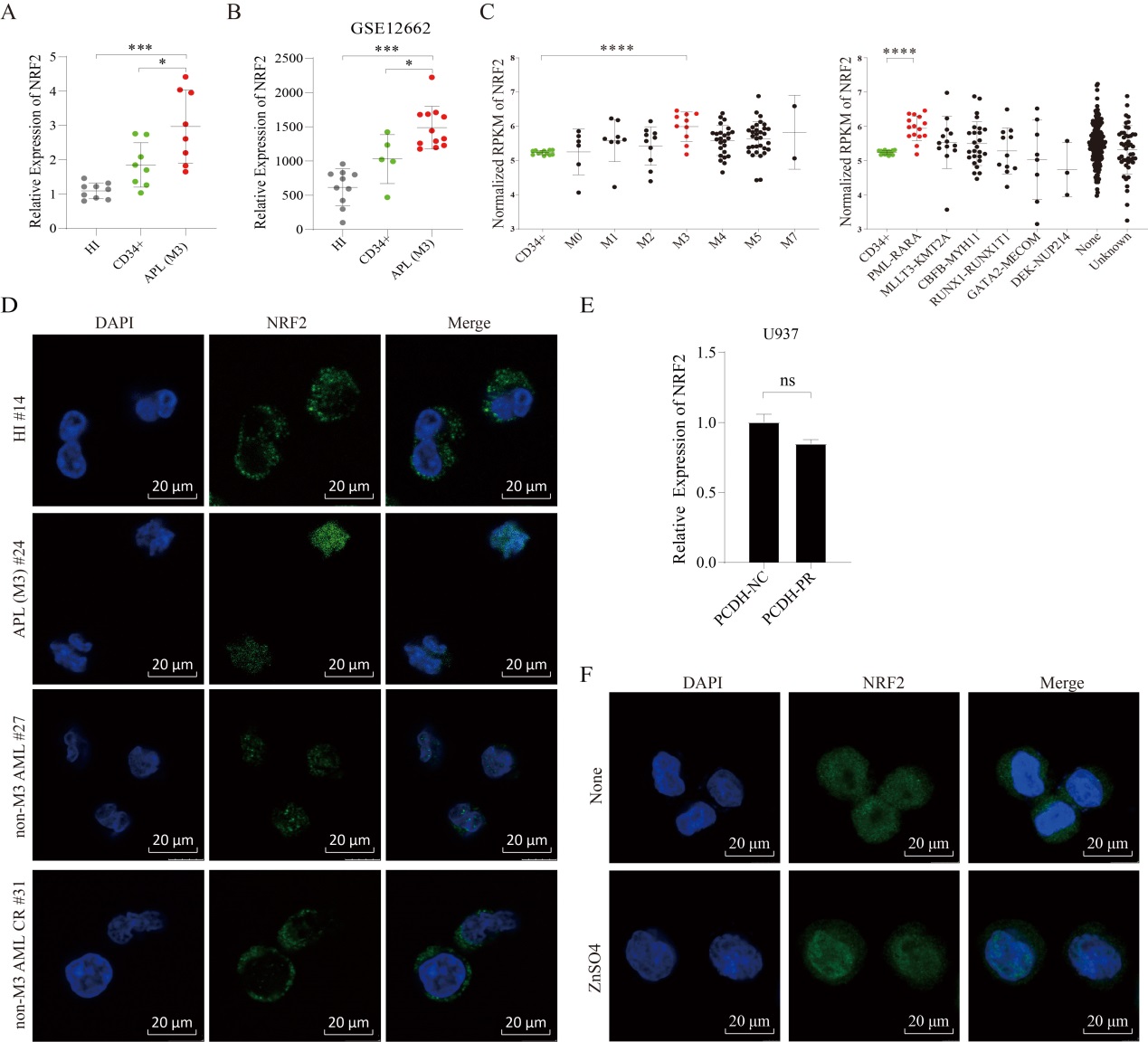
**

**Figure S1.** NRF2 activity is induced by PML-RARα. (A) Comparison of NRF2 expression in PBMCs from HIs (n = 9) compared with APL cells (n = 8) and CD34+ cord blood samples (n = 8). (B) Comparison of NRF2 expression in a GEO database dataset (GSE12662). This comparison revealed that NRF2 expression was significantly elevated in APL samples compared to healthy individuals (HIs) or CD34+ cord blood cells. (C) Expression of NRF2 in the Vizome database. Left panel, NRF2 expression was stratified by FAB Blast Morphology. Right panel, NRF2 expression was stratified by WHO Fusion. (D) Confocal microscopy after staining with an antibody directed against NRF2 (green) in HI, APL (M3), non-M3 AML and non-M3 AML-CR cells. Scale bars, 20 μm. (E) qRT-PCR analysis of NRF2 in U937-PCDH-NC and U937-PCDH-PR cells. (F) Immunofluorescence microscopy after staining with NRF2 (green) and DAPI (blue) demonstrated NRF2 expression in U937-PR9 cells treated with 100 μM ZnSO_4_.

Figure S2

**
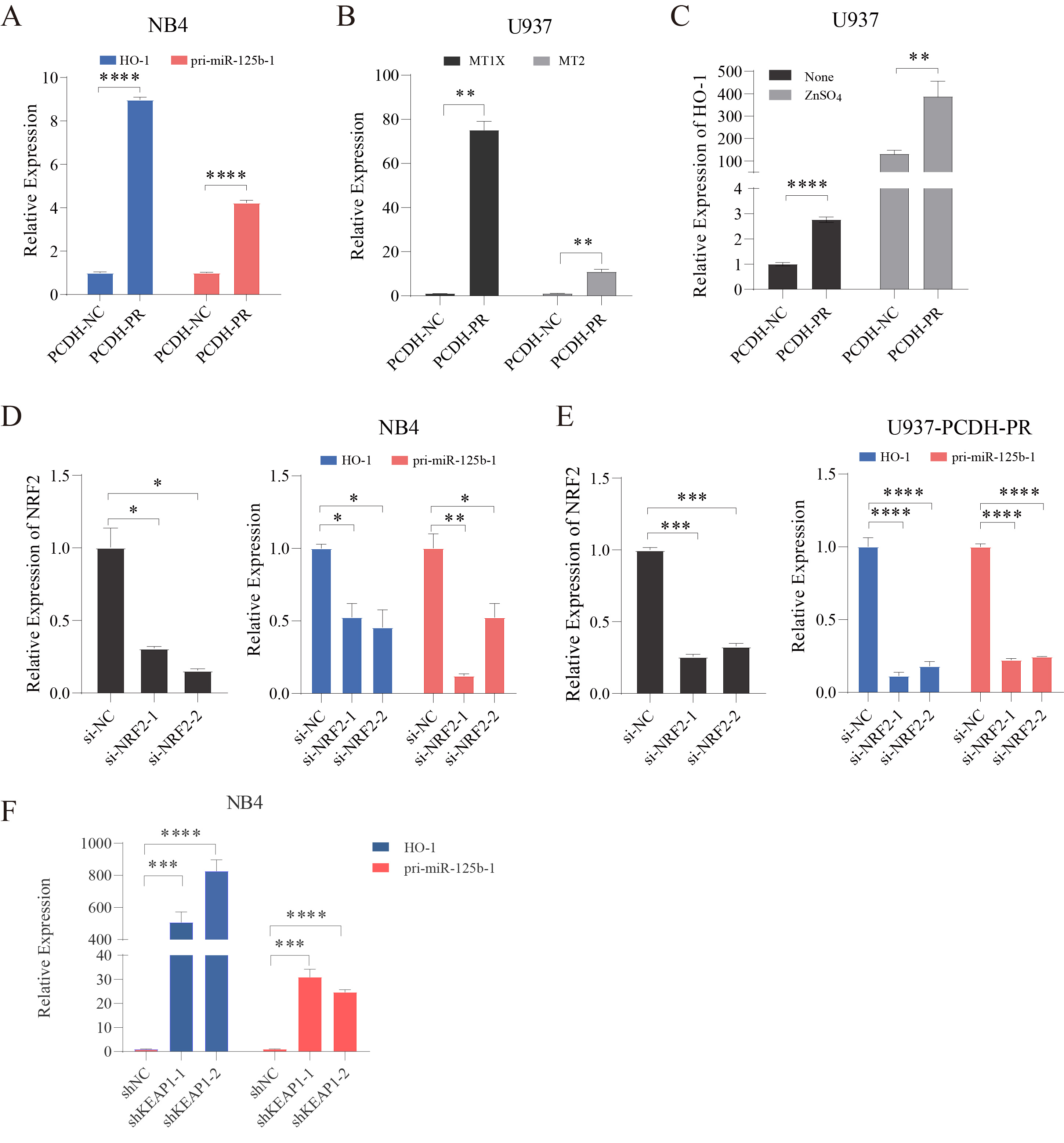
**

**Figure S2.** PML-RARα activates miR-125b-1 expression by NRF2. (A) qRT-PCR analysis of HO-1 and pri-miR-125b-1 in NB4-PCDH-PR cells. (B) qRT-PCR analysis of MT1X and MT2 in U937-PCDH-NC and U937-PCDH-PR cells. (C) qRT-PCR analysis of HO-1 in U937-PCDH-NC and U937-PCDH-PR cells treated with or without 100 μM ZnSO_4_. (D-E) qRT-PCR analysis of NRF2, HO-1 and pri-miR-125b-1 in NB4 and U937-PCDH-PR cells after knocking down the NRF2 gene. (F) qRT-PCR analysis of HO-1 and pri-miR-125b in NB4-shNC and NB4-shKEAP1 cells. Data are presented as the means ± SD from at least 3 independent experiments.

Figure S3

**
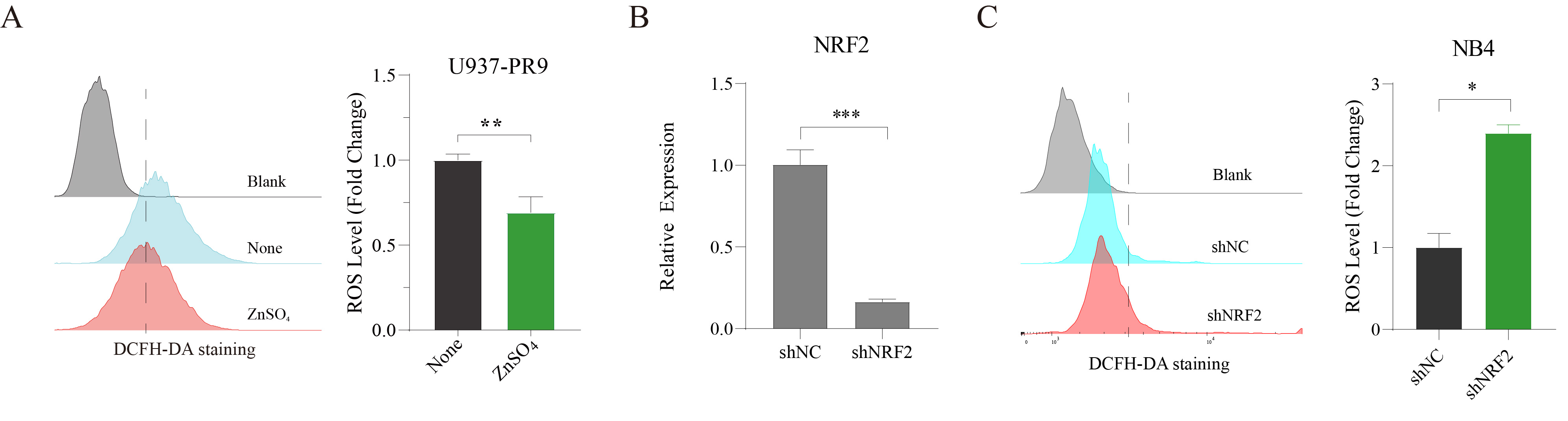
**

**Figure S3.** PML-RARα activates the NRF2 antioxidant program. (A) ROS levels in U937-PR9 cells were analyzed after treated with 100 μM ZnSO_4_. (B) qRT-PCR analysis of NRF2 in NB4-shNC and NB4-shNRF2 cells. (C) ROS levels were assessed in NB4-shNC and NB4-shNRF2 cells by flow cytometric analysis. The graph represents the mean and SD of three independent experiments.

Figure S4


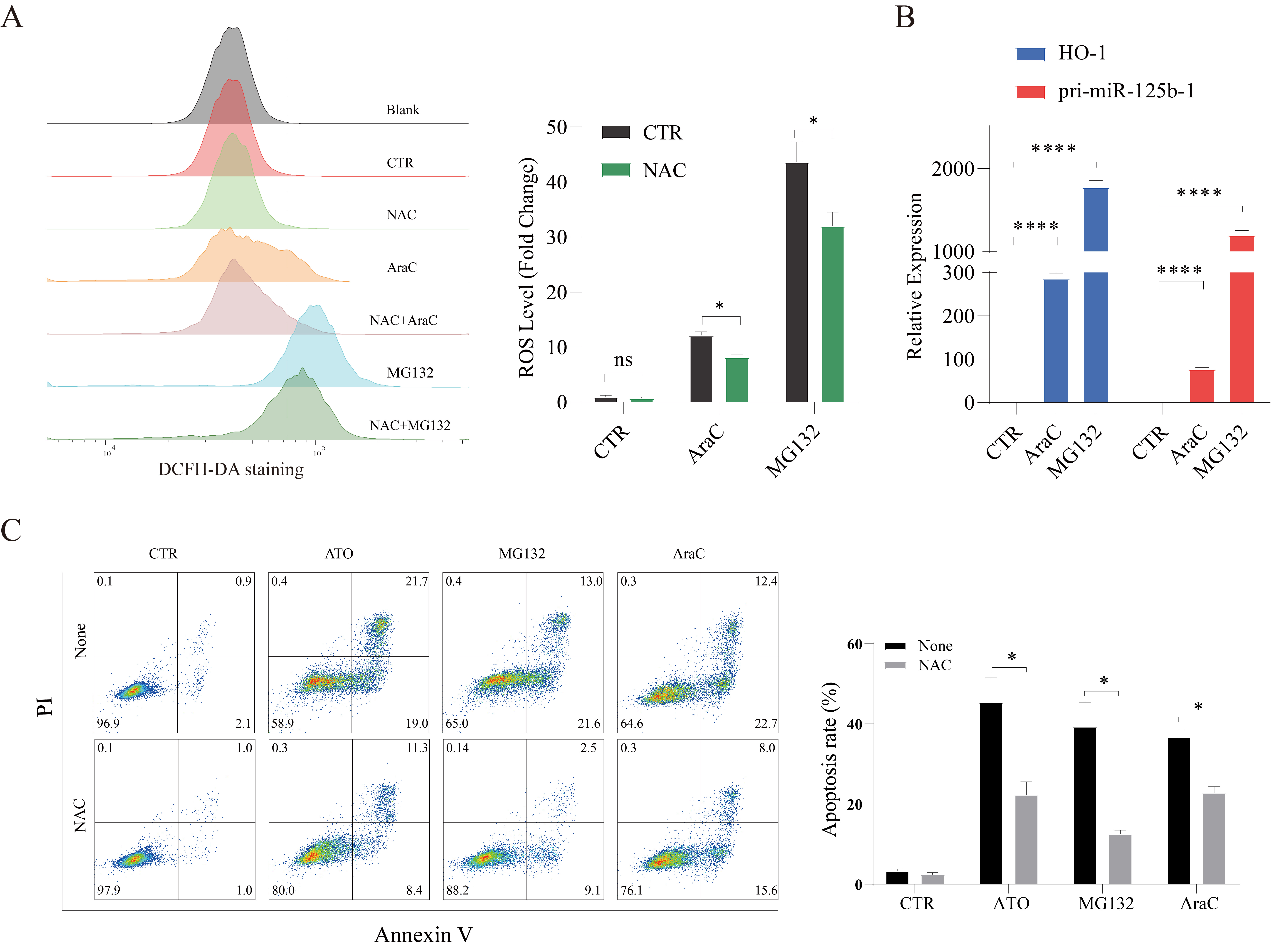


**Figure S4.** MG132- and AraC-mediated ROS induces miR-125b-1 upregulation and apoptosis. (A) The NB4 cells were pretreated with 5 mM NAC for 4 h and then treated with 1 μM MG132 or AraC for 24 h, ROS levels were measured by a DCFH-DA probe. CTR, control. (B) qRT-PCR analysis of HO-1 and pri-miR-125b-1 in NB4 cells. The NB4 cells were treated with 1 μM MG132 or AraC for 24 h. (C) The NB4 cells were pretreated with 5 mM NAC for 4 h and then treated with 5 μM ATO, 0.1 μM MG132 or AraC for 24 h, apoptosis was assessed by flow cytometry analysis.

Figure S5


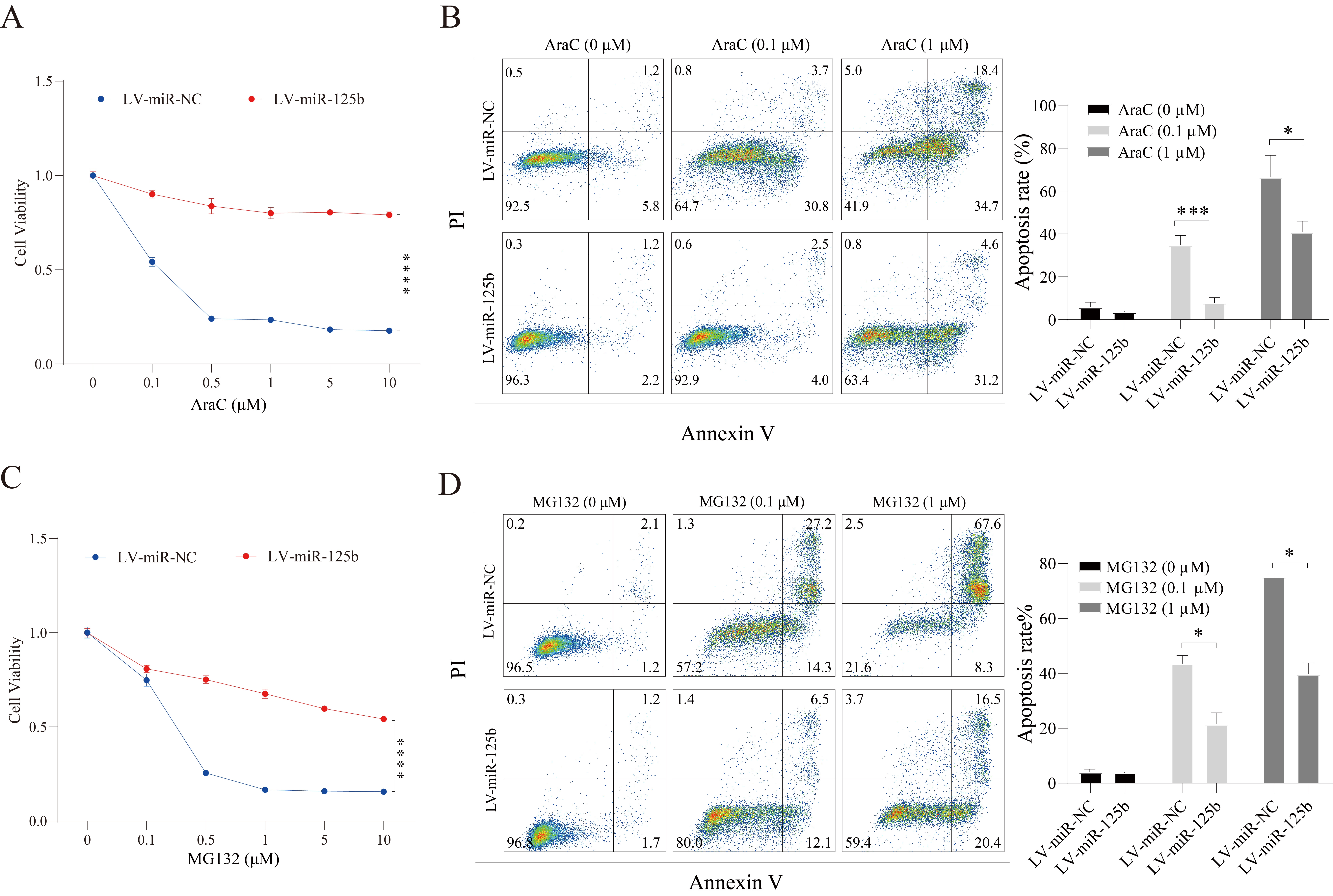


**Figure S5.** miR-125b represses MG132/AraC-induced cytotoxicity in APL. (A) Dose-response curves from cell viability assays of the NB4-LV-miR-NC and NB4-LV-miR-125b cells (AraC, 48 h). A representative image of 3 independent experiments is shown. (B) Flow cytometric analysis of the apoptosis of NB4-LV-miR-NC and NB4-LV-miR-125b cells treated with 0.1/1 μM AraC for 24 h. (C) Dose-response curves from cell viability assays of the NB4-LV-miR-NC and NB4-LV-miR-125b cells treated with the indicated concentration of MG132 for 48 h. (D) Flow cytometric analysis of the apoptosis of NB4-LV-miR-NC and NB4-LV-miR-125b cells treated with 0.1/1 μM MG132 for 24 h. Data are the mean ± SD of three independent experiments.

Figure S6


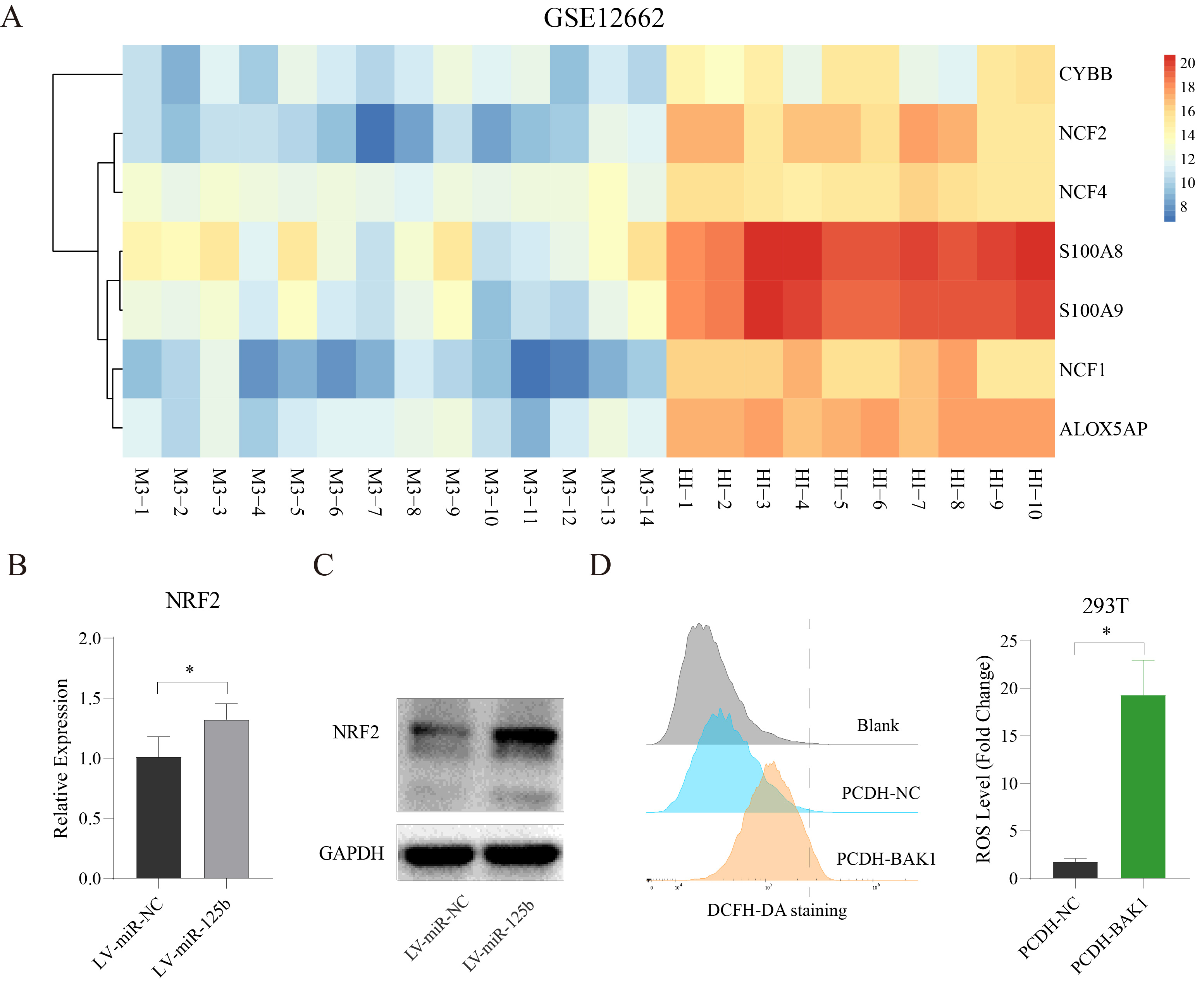


**Figure S6**. Expression of genes involved in managing the level of ROS.

(A) Heatmap plot of ROS production-related enzyme genes in GSE12662. HI, healthy individuals; M3, M3 subtype of AML (APL). (B-C) Changes of NRF2 expression in NB4 cells upon miR-125b overexpression by qRT-PCR and western blot. (D) ROS levels in 293T-PCDH-BAK1 cells were measured by a DCFH-DA probe.
